# Introspective and Neurophysiological Measures of Mind Wandering in Schizophrenia

**DOI:** 10.1038/s41598-020-61843-0

**Published:** 2020-03-16

**Authors:** S. Iglesias-Parro, M. F. Soriano, M. Prieto, I. Rodríguez, J. I. Aznarte, A. J. Ibáñez-Molina

**Affiliations:** 1 Psychology Department, University of Jaén, Jaén, Spain; 2 Mental Health Unit, St. Agustín Universitary Hospital, Linares, Jaén, Spain

**Keywords:** Neuroscience, Human behaviour

## Abstract

Patients with schizophrenia have often been considered to be “in their own world”. However, this casual observation has not been proven by scientific evidence so far. This can be explained because scientific research has usually addressed cognition related to the processing of external stimuli, but only recently have efforts been made to explain thoughts, images and feelings not directly related to the external environment. This internally directed cognition has been called mind wandering. In this paper, we have explored mind wandering in schizophrenia under the hypothesis that a predominance of mind wandering would be a core dysfunction in this disorder. To this end, we collected verbal reports and measured electrophysiological signals from patients with schizophrenia spectrum disorders and matched healthy controls while they were presented with segments of films. The results showed that mind wandering was more frequent in patients than in controls. This higher frequency of mind wandering did not correlate with deficits in attentional, memory or executive functioning. In addition, mind wandering in patients was characterized by a different pattern of Electroencephalography (EEG) complexity in patients than in controls, leading to the suggestion that mind wandering in schizophrenia could be of a different nature. These findings could have relevant implications for the conceptualization of this severe mental disorder.

## Introduction

Research in cognitive neuroscience has extensively addressed cognitive processes and states that are directed towards external stimuli. That is, cognition has been explored in relation to experimental tasks that tapped perception, external attention, memory and so on. Indeed, experimental designs were carefully constructed to minimize the effects of cognition unrelated to the external task, which was considered “noise”^1^. However, the flow of mental contents does not only arise from external inputs; a wide part of cognition is unrelated to the external current environment. Only recently have these internally generated experiences gained the interest of researchers^2^. Internally directed cognition is a frequent experience (it covers between 30% and 50% of the waking brain activity), in which attention disengages from the ongoing external context and focuses on thoughts, mental images, or memories^3^. This type of cognition may appear during the performance of an external task or when an individual is not engaged in any external task (see^4^ for a review). These thoughts, images and feelings away from the external environment have been labelled with several terms, including mind wandering (MW), stimulus-independent thought, or internally directed cognition, among others^5^.

At a neural level, internally directed cognition has been related to the default mode network, a cortical network distributed across the midline of prefrontal cortex, rostral anterior cingulate cortex, posterior cingulate cortex and precuneus^6^. This network was serendipitously discovered in neuroimaging studies, when researchers noticed that specific regions were more engaged during rest than during experimental tasks^7^. Later, it was demonstrated that the default mode network increases its activity when individuals are ruminating about their past, imagining their future, or thinking in their own self, leading to the suggestion that it is responsible for internally directed cognition^8^. MW has also been related to a higher complexity of electrophysiological signals (EEG) than attending to external auditory or visual stimuli in healthy participants^9^. EEG complexity has been considered to be sensitive to the number of independent functional sources in the cortex that contributes to the EEG signal; that is, an increase in the EEG complexity could reflect a decrease in the neural coordination or synchrony related to that signal^10^.

Activity in the default mode network is reduced when individuals perform externally directed tasks^11^. In this line, research has shown that fluctuations between external attention and MW states are governed by dynamic interactions between neural networks. Specifically, activation in the default mode network is anticorrelated to neural networks related to the processing of external stimuli (the salience network and the central-executive network^12)^. This anticorrelation is reflected in perceptual decoupling during MW. That is, when individuals are in MW, the processing of sensory inputs is reduced^13^, and consequently, performance in tasks that tap external attention (such as reading comprehension, sustained attention or working memory) is impaired^14^.

However, MW not only is related to perceptual and attentional functioning but also is associated with emotional states. In an interesting study^15^, MW predisposed patients to a negative mood. They found that people were less happy when their mind was wandering than when they were attending to their present activity. In this line, it has been shown that dysphoria is related to more frequent periods of MW while performing a memory task in non-clinical populations^16^, and a tendency towards MW is negatively correlated with psychological well-being^17^. Although the exact mechanism that underlies the relation between MW and negative mood is unclear, it has been suggested that MW predisposes individuals to rumination and self-centred thinking^18^. Ruminations have an important role in depressive episodes, obsessive-compulsive disorders and schizophrenia. Indeed, rumination has been proposed as a transdiagnostic process that is present in diverse mental disorders^19^. On the other hand, research has shown an association of MW and obsessive-compulsive symptoms and attention deficit hyperactivity disorder symptoms in non-clinical samples^20,21^. With this in mind, it is surprising that the role of mind wandering in the genesis of mental disorders has been so scarcely studied, especially in clinical samples.

In this work, we have explored MW in schizophrenia, under the proposal that the predominance of internally over externally directed cognition would be a core dysfunction in this disorder. That is, we hypothesized that schizophrenia would be characterized by a frequent disengagement of attention from external events that would be accompanied by an excessive focus on the inner world. Hallucinations and delusions would be closely related to this predominance of internally guided cognition, since internal thoughts and images may replace external events, creating a delusional inner world. Although this proposal is new, there is some evidence in its favour. First, abnormal functioning of the default mode network (the brain network associated with MW) has been found in schizophrenia^22^. There are some lines of evidence showing the hyperactivity of this network and aberrant patterns of connectivity, both within this network and between the default mode network and other neural networks^23^. Moreover, patients with schizophrenia do not show the typical deactivation pattern in the default mode network during the performance of an external task^24^. These dysfunctional interactions between networks, for example, between fronto-parietal networks and the default mode network, could be crucial in psychosis, resulting in a disruption in the balance between the processing of external and internal inputs and “blurring the boundaries between the imagination and reality”^8^. Second, as we mentioned before, research has shown an association between MW and ruminations and negative affect^1,18^. There are important similarities between ruminations and hallucinations, which are one of the most relevant psychotic symptoms^25^. Evidence has supported that both hallucinations and intrusions would result from an individual’s difficulty in inhibiting internal intrusive thoughts or images^19,25,26^. Based on these findings, we hypothesize that a predominance of internally guided cognition over externally guided cognition could underlie some of the most relevant psychotic symptoms. Indeed, in a work about MW and schizotypy, which is a relevant risk factor for schizophrenia, it has been demonstrated that a propensity for MW predicts positive, disorganized and paranoid factors of schizotypy^27^. Third, most phenomenological descriptions of schizophrenia have emphasized a dysfunction of the self in the sense that there would be hyperreflexivity (“an exaggerated self-consciousness”, a tendency for focal attention to be directed towards internal sensations or feelings) along with a disturbance in the perception of the external world^28^.

Finally, in the only study that has explored MW in schizophrenia so far, Shin *et al*.^29^ found that patients with schizophrenia showed a higher frequency of MW than controls, and MW frequency was significantly correlated with psychotic symptomatology. However, the results from this study^29^ should be considered with caution since MW was evaluated through a self-report questionnaire. This methodology probably measured the individual’s awareness of their tendency for MW and not MW *per se*. It has been defended that there are two independent components of MW: the disengagement of the mind from external events and the capacity to notice this disengagement^13^. This later component has been named meta-awareness, and research has demonstrated that individuals are only intermittently aware of the fact that their minds have disengaged from the external world. Research exploring the meta-awareness of MW in schizophrenia should have in mind that deficits in metacognition are common in schizophrenia^30^. Metacognition implies self-conscious awareness and reflection about one’s own cognitive events. Then, if the awareness of inner events is a deficit in schizophrenia, we could expect patients to have difficulty in noticing and reporting their minds’ tendency to wander.

This question is related to the difficulties raised in the scientific study of MW. First, MW occurs spontaneously, that is, independent of task instructions. Minds will wander intermittently, and sometimes individuals will not notice it. Second, MW is inherently subjective in nature. Because of this, in MW research, the most employed method has been to ask individuals. The ability to report conscious cognitive states is currently widely accepted^31,32^, and introspective reports are usually used in the scientific study of MW. Introspective measures can be obtained through questionnaires (as in^29^) but can also be provided in an online and straightforward manner through experience sampling probes. In this last method, participants are asked whether they are in MW whenever a probe is presented at intermittent intervals. However, it is recommended to combine subjective and objective data (behavioural and physiological measures)^33^.

In this research, we combined subjective reports with electrophysiological data to explore internally guided cognition in schizophrenia. We have employed experience sampling probes while participants perform a task in an experimental setting. This method is proposed to reflect the actual frequency of MW and not the individual’s metacognitive awareness of MW. In addition, we evaluated cognitive functioning in patients and controls to explore whether cognitive deficits in schizophrenia would be related to the hypothesized differences in MW. It has been shown that individuals with a greater working memory capacity show reduced MW^34^, although this relation is modulated by task demands^35^. MW has also been associated with attentional functioning; it has been found that individuals who have a greater tendency for MW suffer from greater interference from irrelevant stimuli in an external-attention task than those who do not^36^. Since cognitive impairments are well documented in schizophrenia, especially in the domains of attention, executive functions and working memory^25,37–39^, we evaluated whether MW was related to cognitive dysfunctions in patients with schizophrenia.

## Results

### Behavioural data

We analysed the mean differences between groups (SZQ vs CTRL) in the frequency of each reported cognitive state (Audio, Image, Full and MW) in different experimental conditions (Synchrony vs. No Synchrony). We also studied the mean differences between groups (SZQ vs CTRL) in cognitive functioning.

### Internally and externally guided cognitive states

To test significant differences between factors, we selected and conducted a generalized linear model (GENLIN procedure in IBM SPSS Statistics for Windows, Version 19.0)^40^. We did not apply a regular analysis of variance (ANOVA) because the data set we obtained did not meet the assumptions (see Supplementary Information for a detailed explanation). Hence, we performed a GENLIN of the effect of synchronization between visual and auditory inputs (Sync: Synchrony vs. No Synchrony), the cognitive state reported by participants (Cognitive State: Audio, Image, Full, MW) and group (Group: CTRL vs SZQ) on the average frequency of responses.

**Figure 1:**
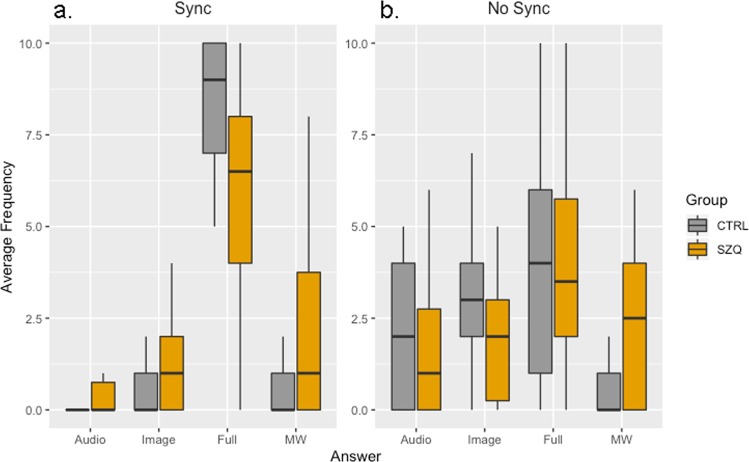
Box plot for average frequency of responses depending on Audio-Image synchronization. Panel a. and b. shows Synchrony and No Synchrony conditions respectively. Each panel shows type of answer reported by participants (Audio, Image, Full, MW) and group (CTRL vs SZQ).

To check for significant fixed effects, we conducted chi-squared Wald tests. All effects were significant (see Fig. 1). Specifically, we found a significant effect of Group [*χ*^2^ (1, *N* = 45) = 6.80, *p* < 0.01]; Cognitive State [*χ*^2^ (3, *N* = 45) = 212.14, *p* < 0.01]; Synchrony [*χ*^2^ (1, *N* = 45) = 23.54, p < 0.01]; Group by Cognitive State [*χ*^2^ (3, *N* = 45) = 19.16, *p* < 0.01]; Group by Synchrony [*χ*^2^ (1, *N* = 45) = 5.88, *p* < 0.05]; Synchrony by Cognitive State [*χ*^2^ (3, *N* = 45) = 69.94, *p* < 0.01]; and, more importantly, Group by Synchrony by Cognitive State [*χ*^2^ (3, *N* = 45) = 9.33, *p* < 0.05].

Post hoc comparisons conducted to analyse the interaction Group by Synchrony by Cognitive State (see Fig. 1), corrected using FRD^41^, showed that in the Synchrony condition, there were no differences between groups in the frequency of Audio episodes (*t* = −1.75, *p* = 0.08) or Image episodes (*t* = −1.55, *p* = 0.12). However, there were significantly more episodes of Full attention in controls than in patients (*t* = −2.07, *p* < 0.05) and more episodes of MW in patients than in controls (*t* = −2.87, *p* < 0.01). On the other hand, in the No Synchrony condition, there were no differences between groups in the frequency of Audio episodes (*t* < 1), either in Image episodes (*t* = 1.81, *p* = 0.07) or in Full attention (*t* < 1). Nevertheless, the frequency of MW episodes was significantly higher in patients than in controls (*t* = −2.61, *p* < 0.01).

### Cognitive functioning

**Figure 2:**
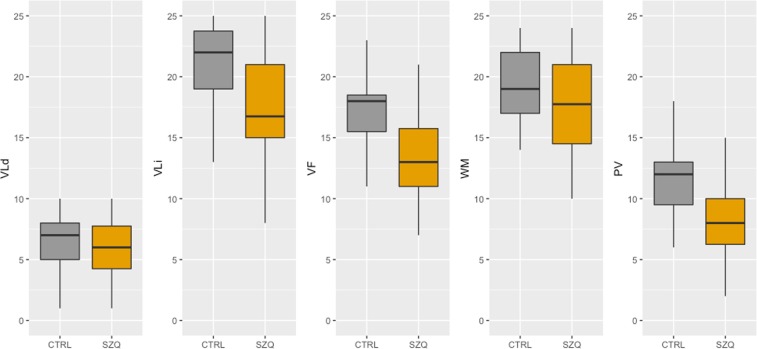
Box plots for SCIP dimensions in each group. VLd: Delayed Verbal Learning, VLi: Immediate Verbal Learning, VF: Verbal Fluency, WM: Working Memory and PV: Processing Speed.

We first performed parametric *t*-tests (all scores were normally distributed) for the scores on the five subtests of the cognitive impairment screening test (SCIP test). *P*-values were corrected using FRD^41^. The results (see Fig. 2) showed that controls performed better than patients in Immediate Verbal Learning (*t* = −2.38, *p* < 0.05), Verbal Fluency (*t* = −3.23, *p* < 0.01) and Processing Speed (*t* = −3.42, *p* < 0.01). No differences were found between patients and controls in Delayed Verbal Learning (*t* = −1.22, *p* = 0.22) or Working Memory (*t* = −1.54, *p* = 0.12).

**Figure 3:**
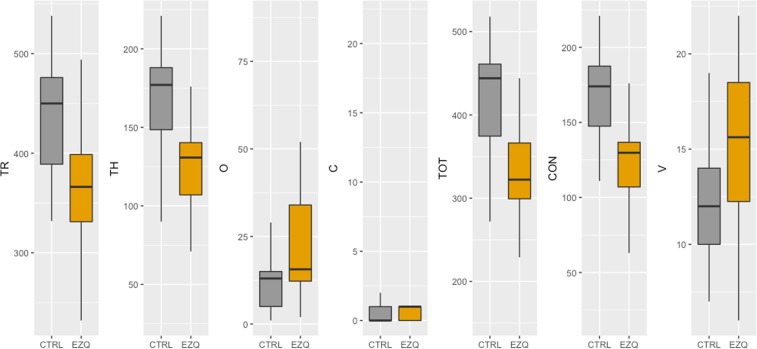
Box plots for D2 test dimensions in each group. TR: total number of responses, TH: total number of hits, O: errors of omission, C: errors of commission, TOT: total test effectiveness, CON: concentration performance and V: variation index.

Second, we performed parametric t-tests for the D2 scores (for errors of omission and errors of commission, we conducted a median test due to non-normality). *P*-values were corrected using FRD^41^. The obtained results (see Fig. 3) showed that controls scored higher than patients in total number of responses (*t* = −3.65, *p* < 0.01), total number of hits (*t* = −3.83, *p* < 0.01), total score (*t* = −3.95, *p* < 0.01), and concentration performance (*t* = −3.58, *p* < 0.01). No differences were found for omissions [*χ*^2^ (1, N = 45) = 2.69, p = 0.10], commissions [*χ*^2^ (1, N = 45) = 0.01, *p* = 0.94] and variation index (*t* = 1.52, *p* = 0.13).

### EEG data analyses

**Figure 4:**
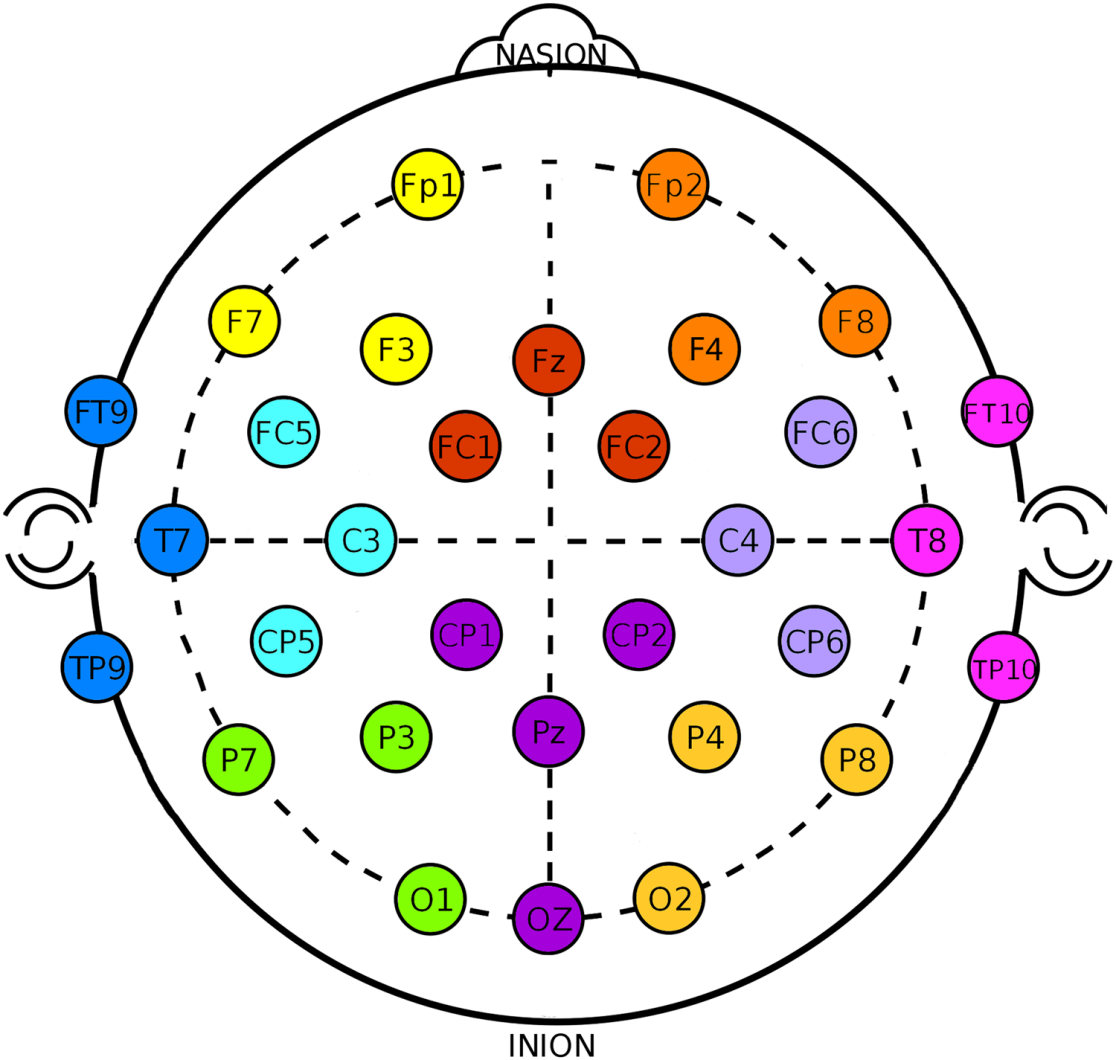
Electrodes and regions of interest used in the study. Each color indicates a different region: Central Left (CL; light blue), Central Right (CR; light purple), Frontal (F; red), Frontal Left (FL; yellow), Frontal Right (FR; orange), Occipital-Parietal (OP; dark purple), Occipital-Parietal Left (OPL; green), Occipital-Parietal Right (OPR; dark yellow), Temporal Left (TL; dark blue), and Temporal Right (TR; pink).

To study EEG complexity, we employed the abovementioned HFD algorithm for the estimation of fractal dimension. Electrodes were grouped in areas of interest (see Fig. 4). We compared values of complexity only in MW and Full Attention cognitive states because of the almost-null frequency of only Image or only Audio responses in some experimental conditions.

We conducted a mixed-effects ANOVA on HFD due to unbalanced data (different number of MW and Full Attention responses across experimental conditions). Analyses were performed in R^42^ using the lmer() function of the lme4 package^43^. Predictor variables included the within-participants factors Synchrony (No Synchrony vs Synchrony), Answer (MW vs Full Attention), Area (CL, CR, F, FL, FR, OP, OPL, OPR, TL, and TR), the between-participants factor Group (SZQ, CTRL), and all interactions. Random intercepts were included for participants. Residuals showed non-important departures from normality, but Levene’s test for homogeneity of variance was significant [*F*(79, 6290) = 4.86, *p* < 0.01]. We used the type III sum of squares because of our unbalanced data. We utilized the restricted maximum likelihood as the estimation procedure because of our relatively small sample^44^, and we used Satterthwaite approximation to estimate denominator degrees of freedom for *F* statistics correction to account for heterogeneous variances^45^.

**Figure 5:**
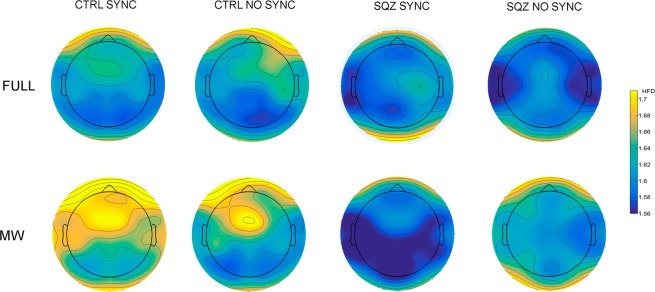
Topographical plots of the HFD distributed on the scalp. The top row shows maps for Full conditions and bottom row MW conditions. Group (CTRL vs SZQ) and synchrony (Sync vs No Sync) are represented in four columns of maps. The colors of the maps indicate the value of HFD on a particular site.

The mixed-effects ANOVA revealed (see Fig. 5) a significant main effect of Answer [*F*(1, 6262.3) = 167.72, *p* < 0.01], reflecting more complexity for MW; a significant main effect of Area [*F*(9, 6248) = 20.92, *p* < 0.01], reflecting higher complexity in frontal areas and lower complexity in occipital areas; and a main effect of Synchrony [*F*(1, 6251.5) = 4.14, *p* < 0.05], reflecting more complexity in the No Synchrony condition. More importantly, we found a significant interaction for Group by Answer [*F*(1,6262.3) = 6.09, *p* < 0.05]; Group by Area [*F*(9,6248) = 15.22, *p* < 0.01]; Answer by Area [*F*(9, 6248) = 4.78, *p* < 0.01]; Group by Synchrony [*F*(1, 6251.5) = 23.72, *p* < 0.01]; Group by Synchrony by Area [*F*(9, 6248) = 3.27, *p* < 0.01]; Group by Answer by Synchrony [*F*(1, 6254.6) = 34.65, *p* < 0.01]; Group by Area by Synchrony [*F*(9, 6248) = 3.27, *p* < 0.01]; and crucially, Group by Answer by Synchrony by Area [*F*(9, 6248) = 2.74, *p* < 0.01]. No significant effect was found for Group [*F*(1,42.3) = 1.41, *p* = 0.24]. No significant interaction was found for Area by Synchrony [*F*(9, 6248) = 1.16, *p* = 0.31]; for Group x Area x Answer [*F*(9, 6248) = 1.75, *p* = 0.07]; or for Answer by Area by Synchrony [*F*(9, 6248) = 1.14, *p* = 0.32].

**Figure 6:**
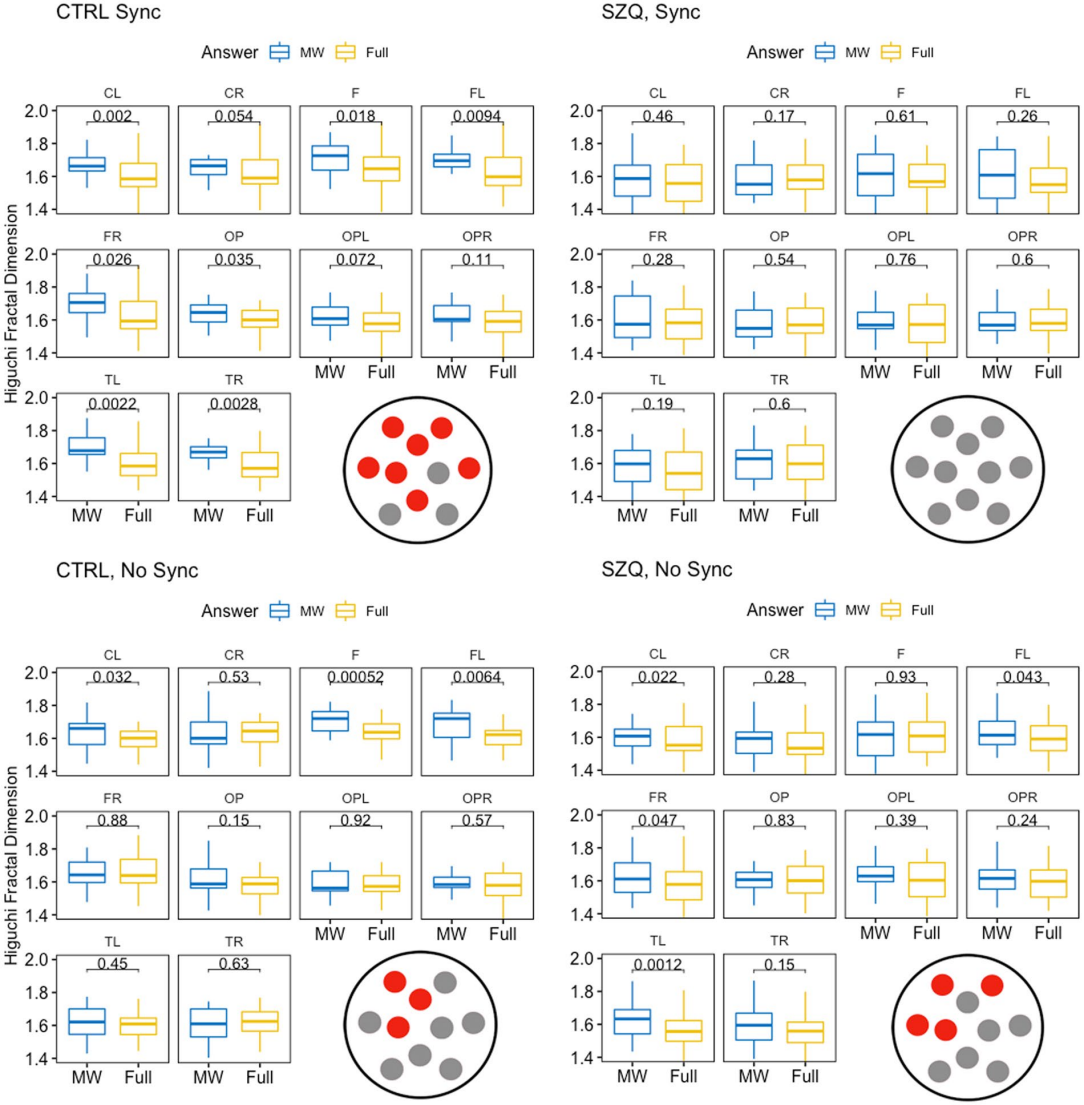
Higuchi Fractal Dimension complexity differences between Full and MW states by group, synchrony condition and areas. *P*-values were corrected using Benjamini-Hochberg procedure. Electrodes and regions of interest used in the study: Central Left (CL), Central Right (CR), Frontal (F), Frontal Left (FL), Frontal Right (FR), Occipital-Parietal (OP), Occipital-Parietal Left (OPL), Occipital-Parietal Right (OPR), Temporal Left (TL), and Temporal Right (TR). Red circles mean significant differences.

Follow-up comparisons were conducted using Group and Synchrony to extract the simple effect of Answer in each area. Wald’s tests were used for the comparisons, and the Benjamini–Hochberg procedure was used to control the increase in type I errors due to the number of comparisons^41^. Comparisons were computed with the R package Phia^46^ using the linear function Hypothesis from the package Car^47^. HFD mean values in each area and *p*-values for MW-Full attention differences are shown in Fig. 6.

In the Synchrony condition, we observed significant differences between MW and Full Attention in most regions (except in the Occipital-Parietal areas) in the control group. That is, in controls, MW was associated with a higher EEG complexity than Full Attention in most brain areas (see Figs. 5 and 6). In contrast, in patients, there were no significant differences in EEG complexity between MW and Full Attention states in any region.

In the condition of No Synchrony, the pattern of EEG complexity was more similar in patients and controls. In the control group, EEG complexity was significantly higher in MW than in Full Attention states in the Frontal, Frontal-Left and Central-Left areas. In the patient group, EEG complexity was significantly higher in MW than in Full Attention states in the Frontal-Right, Frontal-Left, Central-Left and Temporal-Left areas.

In sum, we observed a similar pattern of EEG complexity during MW and Full Attention in both groups in the No Synchrony condition, whereas in the Synchrony condition there were relevant differences in EEG modulation in MW and Full Attention between groups.

### Relationship between the frequency of cognitive states, EEG complexity and other variables

**Figure 7:**
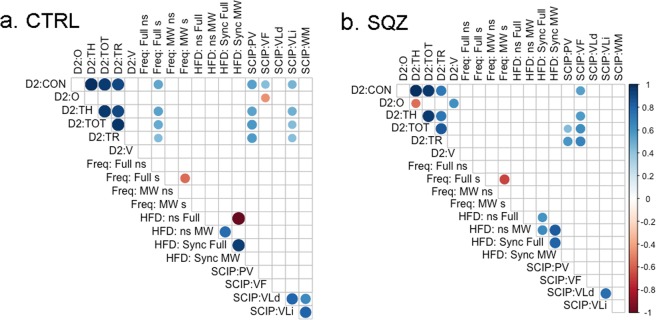
Correlation among the cognitive states, complexity measures and the variables from SCIP and D2 tests. Left panel corresponds to Control group and right panel to Patient group. D2:CON: concentration performance, D2:O: omissions, D2:TH: total number of characters correctly processed (hits), D2:TOT: total score, D2:TR: total number of responses, D2:V: variation index, Freq:Full s: full attention in the synchrony condition, Freq:MW s: mind wandering in the synchrony condition, Freq:Full ns: full attention in the non-synchrony condition, Freq:MW ns: mind wandering in the non-synchrony condition, HFD: Higuchi fractal dimension, SCIP:PV: processing speed, SCIP:VF: verbal fluency, SCIP:VLd: delayed verbal learning, SCIP:VLi: immediate verbal learning, SCIP:WM: working memory.

With the aim of exploring the relationship between cognitive states and cognitive functioning, we performed correlations among the frequency of cognitive states, the EEG complexity (HFD was averaged across all regions) and the SCIP and D2 scores (see Fig. 7).

We found significant positive relationships between the frequency of Full Attention states (in the Synchrony condition) and several attentional indices (four subscale scores of D2) in the control group (see Fig. 7). That is, for controls, a better performance in D2 was related to more frequent episodes of Full Attention states during the experimental task. In the patient group, this relationship was not significant. Regarding EEG complexity, we did not find any significant relationship between HFD values in any condition and behavioural measures.

On the other hand, to explore the relationship between PANSS scores (Total, Positive and Negative symptoms) and the frequency of cognitive states and EEG complexity, Spearman bivariate correlation coefficients were obtained (see Supplementary Information, Fig. SF1). We found no significant relationship between PANSS scores and frequencies of cognitive states or EEG complexity values. In addition, Spearman bivariate correlation coefficients were calculated between age and illness duration and between the frequency of cognitive states and EEG complexity (see Supplementary Information, Fig. SF2). Again, we found no significant relationship.

Finally, we explored the relationship between pharmacological treatment, the frequency of each cognitive state and EEG complexity. To this end, non-parametric tests were first conducted on the frequency of each cognitive state depending on whether participants were taking antidepressants (see Supplementary Information, Table ST1). We also carried out non-parametric tests to study the putative effect of being under mood stabilizers or not on the frequency of cognitive states (see Supplementary Information, Table ST2). We did not find any significant effect of pharmacological treatment on the frequency of cognitive states (see detailed results in Supplementary Information Material). Moreover, non-parametric tests were carried out to study the effect of antipsychotic dose (converted to chlorpromazine equivalents) on Answer frequencies. The results revealed no significant differences in the frequency of cognitive states depending on the antipsychotic dose (see Supplementary Information, Table ST1).

Similar analyses were conducted on EEG complexity measures. The results showed no differences between HFD measures depending on antidepressants (see Supplementary Information, Table ST4) and no differences between HFD measures depending on mood stabilizers (see Supplementary Information, Table ST5) or antipsychotic dose (see Supplementary Information, Table ST6).

## Discussion

Regarding the behavioural results, consistent with our initial hypothesis, we found that patients with schizophrenia showed a significantly higher frequency of MW episodes than controls. These results replicate those found by Shin *et al*.^29^ but with an online measure of MW frequency during an external task. In the Synchrony condition, the greater frequency of MW was accompanied by a decrease in the frequency of full attention responses in patients compared to controls; in contrast, in the No Synchrony condition, patients differed from controls only in MW responses.

Bearing in mind the ample evidence about cognitive deficits in schizophrenia, we were interested in exploring whether this greater frequency of MW could be related to cognitive deficits in the disorder. It has been suggested that MW arises as a failure of executive control^48^. This idea is supported by studies demonstrating that individuals with greater working memory capacity show less frequency of MW episodes during an external task^48^, although this relation is mediated by task demands^35^. Individual differences in MW have also been related to individual differences in susceptibility to external distractions^36^. As patients with schizophrenia present deficits in attention, working memory and executive functions, it would be reasonable to think that the greater frequency of MW in patients with schizophrenia could be related to these deficits. However, we have not found any significant relationship between MW and measures of cognitive functioning. The only significant correlation was between the frequency of Full Attention states and attentional functioning in the control group. It is possible that the small size of the sample prevented us from finding significant correlations between MW and cognitive measures. However, our results are consistent with findings from a study on patients with Alzheimer’s disease^49^. These authors found that patients with Alzheimer’s disease presented more frequent MW than controls, but MW was not correlated with working memory capacity, neither in patients nor in controls. In contrast, they found a significant positive correlation between MW and depression.

It could be argued that differences between patients and controls in the frequency of MW could be caused by difficulties in perceptual integration in schizophrenia. There is some evidence showing that patients with schizophrenia have deficits in multisensory integration^50^. However, our data do not support this interpretation. In the synchronized condition (that is, in the condition in which auditory and visual inputs could be integrated into a holistic perception), the difference between patients and controls in Full Attention responses was not accompanied by differences in Image only or Audio only responses but by differences in MW responses. Specifically, the decrease in the frequency of Full Attention responses in patients compared to controls was accompanied only by an increase in the frequency of MW responses. This suggests that when patients were not attending to the integrated image, it was because they were probably in MW and not attending to a single visual or auditory input.

Regarding EEG signals, MW was characterized by greater EEG complexity than external-attention states. These results are in accordance with results from^9^ with healthy participants. EEG complexity has been related to the integrity of neural connectivity and to the number of distinct cortical generators contributing to a given EEG signal^51^. Hence, complexity would be negatively related to the synchrony of oscillations of these generators. Thus, high levels of complexity in the EEG recording indicate that the neural generators of the signal tend to be widely distributed and desynchronized. In this sense, higher complexity values in MW states would suggest increasingly widely distributed neural nodes oscillating at a lower synchrony. With respect to EEG complexity in patients, previous studies have rendered inconsistent results. While some have found higher EEG complexity in patients with schizophrenia than in healthy controls^52,53^, others have found an opposite pattern^54,55^. These divergences can be related to differences in the complexity measure employed by distinct studies. Our results did not show differences in EEG complexity between patients and controls in general (the main effect of Group was not significant). However, we found a group modulation by electrode sites in which the EEG complexity was lower in patients than in controls in the frontal and temporal areas. This pattern is consistent with previous research that employed HFD as a measure of complexity^56^, whereas higher values of complexity in patients are usually found when Lempel-Ziv complexity is calculated^53,57^.

However, the most interesting finding of our study was the significant interaction between Group, Answer and Area. The analysis of this interaction revealed that, in the Synchrony condition, patients showed no differences in complexity between MW and Full attention; in contrast, the control group displayed an increase in EEG complexity in MW compared to Full Attention states (consistent with findings from previous research^9^).

This novel finding, combined with the greater frequency of MW in patients than in controls, allows us to draw some tentative conclusions about MW in schizophrenia. On the one hand, evidence has shown that the default mode network is hyperactivated in schizophrenia, whereas the structural connectivity of the network is reduced (especially in the prefrontal regions of the network). In addition, it has been demonstrated that in schizophrenia, there is no deactivation of the default mode network during an external task. MW episodes have been related to the activation of the default mode network and to an increase in the complexity of the EEG. According to the neurocomputational model proposed by Ibáñez-Molina and Iglesias-Parro^10^, when the default mode network is activated, EEG complexity increases in the presence of external stimulation. When the DMN is deactivated, complexity decreases.

Considering this evidence, we propose that in schizophrenia, the lack of the deactivation of the default mode network during external tasks^24^ would give rise to an increase in MW episodes, but the less cohesive functioning would be related to a different nature of MW in patients, reflected in the similarity between their MW and externally directed states in terms of EEG complexity. That is, patients with schizophrenia present an increased frequency of MW states, but these states, as they arise from a disconnected network, are characterized by lower EEG complexity than MW in healthy participants. This seems to imply that MW and external-attention states would not be so differentiated in patients with schizophrenia as in healthy participants.

This finding raises an interesting question about a possible different nature of MW states in patients. Given the pioneering nature of this research, we have not differentiated between different types of MW. However, internally guided cognitive states (as well as externally guided cognitive states) differ along multiple dimensions^5^: content, intentionality, and relation with the self, among others^21^. The different pattern of EEG complexity in MW states in patients and controls and the finding of a higher frequency of MW in patients, suggest that there can be important differences in the nature and quality of MW between patients and controls.

Finally, we did not find any relevant relationship between cognitive functioning and symptoms that could explain differences in MW in schizophrenia. The only significant relationship was between the frequency of Full Attention states and attentional scores in the control group. It is reasonable to expect that better attentional functioning would facilitate attention to both images and sound in synchronized films. However, we did not find this relationship in the patient group, and MW frequency was not related to attentional scores in any group. However, it is possible that we were not able to capture relevant relations because of the small sample size.

In sum, two interesting findings have emerged from this research work. First, as expected, we found a higher frequency of MW in patients than in controls. Second, patients failed to show an increase in EEG complexity, which was evident in controls. We speculate that the absence of the deactivation of the default mode network during external tasks^24^ would give rise to an increase in MW episodes, but the less cohesive functioning would be related to a different nature of MW in patients. These explanations are exploratory; future studies should explore the specific dimensions of MW that are predominant in schizophrenia and related mental disorders.

## Method

### Participants

In this study participated 22 adults from the Mental Health Day Unit at the St. Agustín Universitary Hospital (Spain) (Group: SZQ). The inclusion criteria for participation were an ICD-10 diagnosis of schizophrenia (F20, 19 patients), psychotic disorder (F23, 1 patient), or schizoaffective disorder (F25, 2 patients). The diagnosis of participants was made by the psychiatrist or clinical psychologist in charge of the patient. The age range was between 23 and 53 years old (M = 36.54; SD = 10.23). Out of the 22 participants, 7 (30.4%) were female. All participants were right-handed. Regarding educational level, 5 had primary education, 13 participants had secondary education, and 7 had higher education. Their mean illness duration was 14.68 years (SD = 9.53, min = 1, max = 35). All patients were under antipsychotic treatment: 21 were on typical antipsychotics, and 1 was on atypical antipsychotics. Ten participants were taking only one antipsychotic, and 12 participants were taking two or more antipsychotics. Eleven participants were taking different combinations of antipsychotics, together with antidepressants (3) or with mood stabilizers (11), whereas 11 participants were on only antipsychotics. We converted all antipsychotic doses to chlorpromazine equivalents (M = 676.14 mg, SD = 359.43 mg). Detailed information about medication effect analyses can be found in section 4 of the Supplementary Information.

For the control group, 23 adults were recruited from the University of Jaén, staff from St. Agustín Hospital and an adult school from Jaén (Group: CTRL). The age range was between 23 and 57 years (M = 38.87 years old; SD = 11.86 years old). Out of the 22 participants, 4 (18.2%) were female. Twenty-one participants were right-handed, whereas 2 were left-handed. Regarding educational level, 2 participants had primary education, 14 participants had secondary education, and 7 participants had higher education. There was no significant association between Gender and Group [*χ*^2^(1, *N* = 45) = 0.91, *p* = 0.33] or between Group and Educational level [*χ*^2^ (2, *N* = 45) = 2.12, *p* = 0.34]. There were no significant differences between Groups depending on Age (*t* = 0.70, *p* = 0.48). Exclusion criteria for both groups were a concurrent diagnosis of neurological disorder, a concurrent diagnosis of substance abuse, a history of developmental disability, an inability to sign informed consent or vision disorders (vision disorders that, although corrected by glasses or contact lenses, suppose a loss of visual acuity, e.g., cataracts) or auditive disorders (unless they were corrected by hearing aids). In addition, an exclusion criterion for the control group was the diagnosis of a mental disorder (according to verbal reports from participants). All participants gave their written informed consent according to the Declaration of Helsinki, and the Ethics Committee on Human Research of the Hospital (*Comité de Ética de la Investigación de Jaén*) approved the study.

### Materials

#### Experimental task

The experimental task consisted of a presentation of a series of videoclips. We selected an initial set of segments extracted from 6 non-blockbuster Spanish films. All segments contained quiet scenes with dialogues. We separated the audio and Image tracks and factorially combined them. We obtained a total of 36 videoclip segments: 6 of them were the original audio-visually synchronized clips and 30 were clips in which the audio and image information belonged to different movies (not synchronized). We constructed 6 counterbalanced versions of 5 videos composed of two audio-visually synchronized videoclips, two non-synchronized videoclips, and one clip for practice. These versions were constructed in a way that all films appeared with the same frequency as synchronized or not synchronized across participants, and importantly, no film was used for different clips in the same version. Participants were randomly assigned to one of these versions. Thus, each participant was presented with a combination of four videos, each of 5-min length: in two of them, visual and auditory information matched, whereas in the other two, visual and auditory information did not match (for example, the image sequence of a 5-min-length clip of a Spanish film was presented on the screen while the sound of a 5-min-length piece of a different Spanish film was presented through the loudspeakers). We made sure that participants were not familiar with the films by asking them at the end of the task. Only one patient reported vaguely remembering one of the movies.

#### EEG equipment

EEG data were obtained and recorded with a 32-channel BrainAmps EEG/ERP Recorder through Vision Recorder software.

#### Cognitive evaluation

The Spanish version of the Screen for Cognitive Impairment in Psychiatry (SCIP-S^58^) was employed to measure cognitive impairment. The SCIP-S is a brief screening tool designed to assess cognition in clinical samples and has demonstrated reliability and concurrent validity with extensive neuropsychological batteries^59^. The SCIP-S is composed of five subtests and can be completed with pencil and paper in approximately 15 minutes. It provides an individual score for each subtest of working memory, immediate and delayed verbal learning, verbal fluency, and psychomotor speed as well as a composite global score.

In addition, as we wanted to evaluate the specific relationship between MW and attentional functioning, we employed the Spanish version^60^ of the **D2 Test of Attention**^61^, which is a paper-and-pencil cancellation test where individuals have to scan for target items among distracters. It provides a number of scores: total number of responses (TR); total number of hits (TH); omitted elements (O); commissions (that is, the number of irrelevant elements marked) (C); total test effectiveness (TOT) (that is, TR − (O + C)); concentration index (CON) (that is, TH-C); the line with a greater number of tried elements (TR+); the line with a lower number of tried elements tried (TR−); and variation index or difference (V) (that is TR+− TR−).

### Symptoms scale

The Spanish version^62^ of the Positive and Negative Syndrome Scale (PANSS^63^) was used to assess psychopathology. It can be divided into three subscales: the positive subscale of 7 items (M = 14, SD = 6.08), the negative subscale of 7 items (M = 18.85, SD = 7.60), and the general psychopathology subscale of 16 items (M = 32.41, SD = 9.33).

### Procedure and data recording

The research was carried out in two sessions. In the first session, participants sat in a laboratory room at the hospital, in front of a computer screen, approximately 70 cm away from the centre of the screen. Then, the experimenter proceeded to place the cap montage of 31 active electrodes in the 10–20 system with positions FP1, FP2, F7, F3, Fz, F4, F8, FT9, FC5, FC1, FC2, FC6, FT10, T7, C3, C4, T8, TP9, CP5, CP1, CP2, CP6, TP10, P7, P3, Pz, P4, P8, O1, Oz and O2. All electrodes were referenced to both mastoids, and impedances were kept below 5 kOhm. Signals were recorded with an AD rate of 500 Hz.

After the cap montage was in place, we gave instructions to participants about the experimental task. We intended to create an experimental setting that simulated natural situations in daily life; to this end, we emphasized different points. First, we informed participants that, in daily life, when we are presented with a film, we occasionally get distracted and think on “our own things”, or we attend only to a conversation or image from the film that captures our attention. After that, we told participants that they were going to be presented with several segments of films, and after each segment, we were going to ask whether they were attending to the image, to the sound, to both the image and the sound, or to any other content they had in their minds. We emphasized that there were no correct responses; we were interested only in how minds usually work. Because of that, it was not necessary that they made an extra effort to pay attention, only that they attended to the films as they would if they were watching them in their own home. In addition, they were told that in some films, the image and sound did not belong to the same movie (not synchronized condition).

Every 60 ± 10 s, the video paused and participants were asked, by a message on the screen, to verbally report if they were attending to the visual inputs, the auditory inputs, or both of them or if they were in an MW state. The researcher, who remained outside the participant’s visual field, registered the verbal response by introducing a numeric code in the EEG recording. Verbal reports were allowed up to 10 s; then, the message disappeared, and the video continued from the same time point where it was paused. The experimental video presentation was preceded by some practice videos to ensure participants understood the instructions.

In the second session, participants underwent the cognitive evaluation. After that (only in the case that participants were patients), a clinician administered the PANSS, and some additional data (age, gender, educational level, pharmacological treatment, illness duration) were collected.

### EEG Data analysis

Data processing was performed with the Brain Vision Analyzer software EEGLAB^64^ and MATLAB^65^. We applied a bandpass filter with cut-off frequencies of 1 and 30 Hz. For each participant, we selected 20 clean EEG segments, each with a length of 50 s. All of them were labelled according to the introspective answer of participants (Image, Audio, MW and Full attention) and the type of synchronization of the videoclip during the registration of the EEG segment (Synchronized or Not Synchronized). Blinks and other artefacts were extracted using infomax ICA^66^. ICA components with artefacts were selected by visual inspection of the scalp topography, power spectra and raw activity from all components. Once all noisy components were selected, they were extracted from the original signals. The resulting EEGs were used as inputs for a custom MATLAB script developed to obtain Higuchi’s fractal dimension (HFD^67^) as an indicator of signal complexity. HFD is a measure of self-similarity and irregularity that can be easily applied in the time domain (time series). As a consequence, it ranges from 1 (simple signals as a sinusoidal wave) to 2 (a signal that nearly fills all Euclidean 2D space) (see^9^). In the experiment we present here, we calculated a single HFD estimation for each segment using a sliding window procedure. Specifically, we used a sliding procedure with a window length of 2000 ms and 90% overlap. Finally, the HFD was estimated for each segment by averaging all values in the sliding window.

## Supplementary information

Supplementary information

## Data Availability

The data that support the findings of this study are available from Synapse.org but restrictions apply to the availability of these data, so are not publicly available. Data are however available from the authors upon request and with permission of Synapse.org.
