# Supplementary material for: Introspective and Neurophysiological Measures of Mind Wandering in Schizophrenia
Source: Sci Rep. 2020 Mar 16;10:4833. doi: 10.1038/s41598-020-61843-0 (PMC7076020; doi:10.1038/s41598-020-61843-0)
Supplement: Supplementary file 1 — Supplementary information [file 41598_2020_61843_MOESM1_ESM.pdf]

Introspective and Neurophysiological Measures of Mind Wandering in Schizophrenia.

Iglesias-Parro, S.*[1], Soriano, M.F.[2], Prieto, M.[1], Rodriguez, I.[1], Aznarte, J.I.[2] & Ibáñez-Molina, A.J.[1].

[1]Psychology Department, University of Jaén.

[2]Mental Health Unit, St. Agustín Universitary Hospital, Linares (Jaén).

Author for correspondence: Iglesias-Parro, Sergio (siglesia@ujaen.es)

Departamento de Psicología
Universidad de Jaén
Paraje las Lagunillas s/n
Jaén, 23071 (Spain)

Supplementary Information

Statistical model for Behavioral data analyses

Due to no-normality of the data and because count data with many zero values cannot be made normal by transformation[1], as well as due to the observed heterogeneity of variances, we used a Generalized Linear Model (GENLIN procedure in IBM SPSS Statistics for Windows, Version 19.0, IBM Corp., 2010). Parameters were estimated by penalized quasilikelihood (PQL) method[2]. Because we were working with count data, we selected the Poisson distribution as target distribution (the underlying error distribution for the dependent variable). In order to linearize the relationship between dependent variable and predictors, we selected the logarithm as the link function.

After that, the full factorial model was tested. For parameter signification test, we used the Satterthwaite approximation for the error degrees of freedom in *t*-test[3]. Results showed a significant increase on fit with the full model when was compared with the intersection only model [$\chi^2(15, N=45) = 589.82$, $p < .01$]. However, the Poisson model implies equality of the variance and the mean (equidispersion) and there seems to be signs of overdispersion (deviance=651.35, *df*=344, ratio=1.83). In order to check statistically for overdispersion, we run an auxiliary OLS regression on alpha parameter (see [4] for details). Obtained results ($z = 5.93$, $p < .01$) indicated significant overdispersion. To deal with the overdispersion we re-scaled the dependent variable by the inverse of the deviance/df ratio. The scaling does not alter the parameter estimation but it adjusts the standard error making the statistical test more conservative. Obtained results after scaling the dependent variable showed a significant increase on fit with the full model when compared with intersection [χ^2 (15, $N=45$) = 312.07, $p < .01$]. After re-scaling, no overdispersion signs were found (deviance=344.63, *df*=344, ratio=1.002).

Supplementary Figure SF1. Relationship between PANSS scores and frequency of cognitive states.

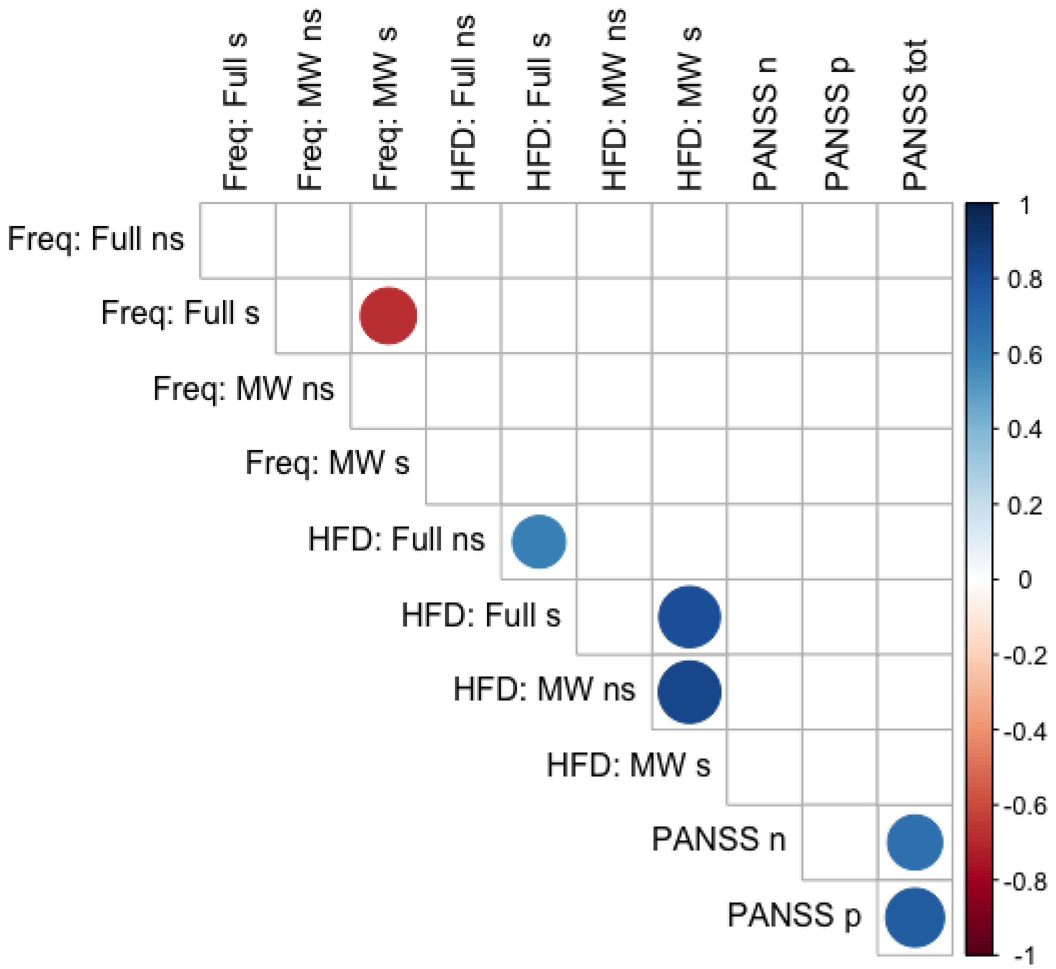

Figure SF1. Spearman bivariate correlation coefficients between PANSS scores (PANSS n for negative symptoms, PANSS p for positive symptoms and PANSS tot for total score on PANSS), frequency of cognitive states (Freq: Full no synchrony, Freq: Full synchrony, Freq: MW no synchrony, Freq: MW synchrony) and EEG complexity measures (HFD: Full no synchrony, HFD: Full synchrony, HFD: MW no synchrony, HFD: MW synchrony). Only significant correlations are signaled.

Supplementary Figure SF2. Relationship between age and illness duration and frequency of cognitive states.

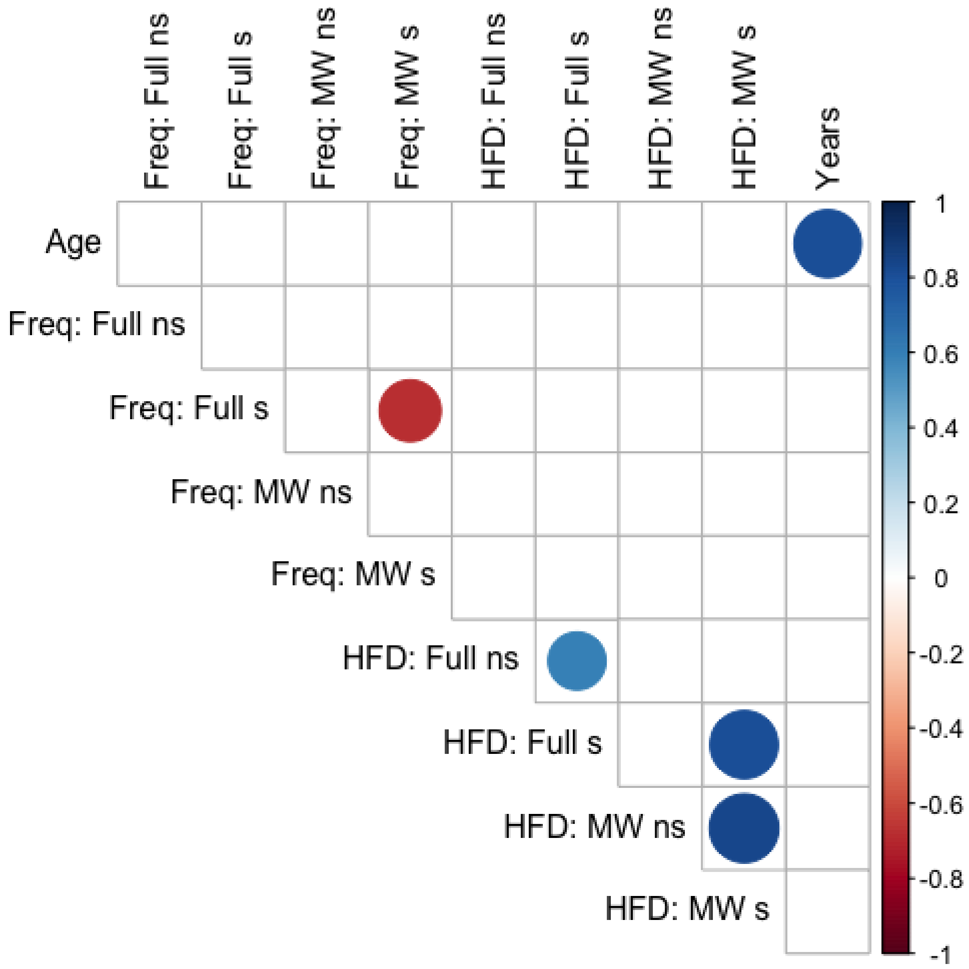

Figure SF2. Spearman correlation coefficients between age (Age) and illness duration (Years), frequency of cognitive states (Freq: Full no synchrony, Freq: Full synchrony, Freq: MW no synchrony, Freq: MW synchrony) and EEG complexity measures (HFD: Full no synchrony, HFD: Full synchrony, HFD: MW synchrony, HFD: MW no synchrony). Only significant correlations are signaled.

Obtained results showed no relevant relationships between frequency of cognitive states, EEG complexity measures and age-related variables.

Supplementary Tables ST1 to ST6. Medication effects.

We explored the relationship between pharmacological treatment and frequency of each cognitive state. First, non-parametric tests were conducted on frequency of each

Cognitive State depending on whether participants were taking antidepressants or not. Results of Mann-Whitney tests are summarized in supplementary Table ST1. Results revealed no significant differences for Answer frequencies (Audio, Image, MW and Full attention) depending on antidepressants.

Table ST1. Mann-Whitney tests values (U) and *p*-values for Answer frequencies depending on antidepressants (No vs Yes) for Answer frequencies.

	Audio	Image	MW	Full
U	26.00	21.00	13.50	26.50
P-value	.80	.46	.14	.84

We also carried out non-parametric tests to study the putative effect of being under mood stabilizers or not on Answer frequencies. Results of Mann-Whitney tests are summarized in supplementary Table ST2. Results revealed no significant differences for Answer frequencies (Audio, Image, MW and Full attention) depending on mood stabilizers.

Table ST2. Mann-Whitney tests values (U) and *p*-values for Answer frequencies depending on mood stabilizers (No vs Yes) for Answer frequencies.

	Audio	Image	MW	Full
U	49.00	58.50	49.00	60.00
P-value	.44	.89	.44	.97

Non-parametric tests were carried out in order to study the putative effect of antipsychotic dose on Answer frequencies. To do this, we converted all antipsychotic doses to chlorpromazine equivalent. Next, we assigned the participants to one of two groups (High dose or Low dose) depending on whether they scored over or above chlorpromazine equivalent Mean on this sample. Results of Mann-Whitney tests are summarized in supplementary Table ST3. Obtained results revealed no significant differences for Answer frequencies (Audio, Image, MW and Full attention) depending on chlorpromazine equivalent doses.

Table ST3. Mann-Whitney tests values (U) and *p*-values for Answer frequencies depending on mood stabilizers (No vs Yes) for Answer frequencies.

	Audio	Image	MW	Full
U	44.00	51.50	34.00	53.50
P-value	.27	.54	.08	.64

We also explored the possible relationship between pharmacological treatment and EEG complexity measures. First, non-parametric tests were conducted on HFD of each Cognitive State depending on whether participants were taking antidepressants or not. Results of Mann-Whitney tests are summarized in supplementary Table ST4. Results revealed no significant differences for HFD measures (MW sync, Full sync, MW no sync, Full no sync) depending on antidepressants.

Table ST4. Mann-Whitney tests values (U) and *p*-values for HFD depending on antidepressants (No vs Yes) for EEG complexity measures.

	MW sync	Full sync	MW no sync	Full no sync
U	6.00	14.00	17.00	15.00
P-value	.27	.61	.88	.88

We also carried out non-parametric tests to study the possible effect of being under mood stabilizers or not EEG complexity measures. Results of Mann-Whitney tests are summarized in supplementary Table ST5. Results revealed no significant differences for HFD measures (MW sync, Full sync, MW no sync, Full no sync) depending on mood stabilizers.

Table ST5. Mann-Whitney tests values (U) and *p*-values for HFD depending on mood stabilizers (No vs Yes) or EEG complexity measures.

	MW sync	Full sync	MW no sync	Full no sync
U	24.00	46.00	27.00	30.00
P-value	.94	.76	.99	.34

Finally, non-parametric tests were carried out in order to study the putative effect of antipsychotic dose on EEG complexity measures. We assigned the participants to one of two groups (High dose or Low dose) depending on whether they scored over or above chlorpromazine equivalent Mean on this sample. Results of Mann-Whitney tests are summarized in supplementary Table ST6. Obtained results revealed no significant differences for HFD measures (MW sync, Full sync, MW no sync, Full no sync) depending on chlorpromazine equivalent doses.

Table ST6. Mann-Whitney tests values (U) and *p*-values for HFD depending on mood stabilizers (No vs Yes) or EEG complexity measures.

	MW sync	Full sync	MW no sync	Full no sync
U	21.00	45.00	22.50	34.00
P-value	.53	.61	.22	.41

References

1. Bolker, B. M. *et al.* Generalized linear mixed models: a practical guide for ecology and evolution. *Trends Ecol. Evol.* **24**, 127–135 (2009).
2. Schall, B. Y. R. Estimation in generalized linear models with random effects. *Biometrika* **78**, 719–727 (1991).
3. Satterthwaite, F. E. An Approximate Distribution of Estimates of Variance Components Published by : International Biometric Society. *Biometrics Bull.* **2**, 110–114 (1946).
4. Cameron, A. C. & Trivedi, K. P. Microeconometrics: methods and applications. *Cambridge Univ. Press* (2005).
